# PFO Leading to Concomitant Platypnea-Orthodeoxia Syndrome and Stroke

**DOI:** 10.1016/j.jaccas.2024.102439

**Published:** 2024-08-07

**Authors:** Benjamin Mancini, Francois Kaleta, Eric Zimmerman, Gianna Daflissio, Samer Muallem, Eric Chan, Mark Kozak, Ryan Wilson

**Affiliations:** a Penn State College of Medicine, Hershey, Pennsylvania, USA; b Penn State Heart and Vascular Institute, Hershey, Pennsylvania, USA; c Penn State Hershey Medical Center, Hershey, Pennsylvania, USA

**Keywords:** bubble echocardiography, hypoxemia, imaging, occluder, stroke

## Abstract

Platypnea-orthodeoxia syndrome is a rare entity seen in patients with patent foramen ovale, characterized by dyspnea and arterial desaturation in the upright position. We describe a case of a patient who presented with cryptogenic stroke, evidence of right-to-left interatrial shunting, and refractory hypoxemia necessitating closure of his patent foramen ovale.

## Graphical Abstract

**Figure:**
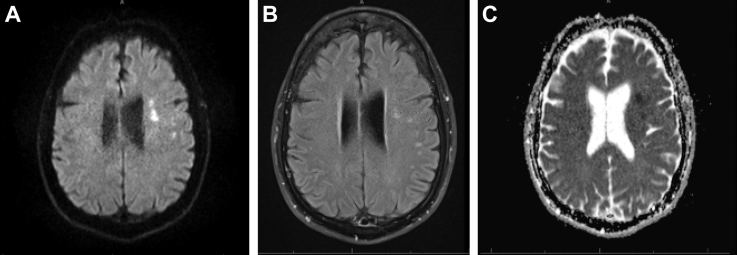

## History of Presentation

The patient is a 68-year-old male with hyperlipidemia who presented to the hospital with right-sided facial droop and word-finding difficulty. The neurology team evaluated the patient in the emergency department and workup confirmed an acute ischemic stroke. However, the patient did not qualify for thrombolytic therapy as the last known functional baseline was approximately 24 hours before presentation.

**Learning Objectives**

• To understand the epidemiology and potential complications of PFO.
• To understand the appropriate workup and management of symptomatic PFO.
• To appreciate the pathologic anatomic mechanisms contributing to POS in the context of PFO and its relationship to the risk of embolic stroke.

The physical examination was notable for a right facial droop and expressive aphasia. National Institutes of Health Stroke Scale (NIHSS) score on admission was 3. Vitals signs demonstrated new hypoxemia with O_2_ saturation approximately 80% on room air. The patient subsequently required high-flow nasal cannula to maintain an oxygenation level >90%. It was noted that the hypoxemia was positionally dependent; however, the patient denied any shortness of breath or syncope before presentation. The remainder of the physical examination was unremarkable.

## Past Medical History

The past medical history is only notable for hyperlipidemia.

## Differential diagnosis

The differential diagnosis includes an intracardiac shunt, intrapulmonary shunt, pneumonia, chronic thromboembolic pulmonary hypertension, and cardiac arrythmia.

## Investigations

**Figure 1:**
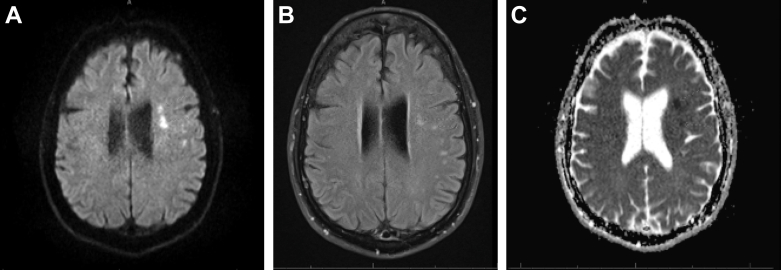
Magnetic Resonance Imaging (A) Magnetic resonance imaging (MRI) diffusion-weighted imaging. (B) MRI fluid-attenuation inversion recovery. (C) MRI apparent diffusion coefficient. MRI revealed multiple areas of cerebral infarction including the left operculum, insular cortex, and left internal capsule with subtle hemorrhagic conversion.

A computed tomography scan of the head without contrast revealed no acute intracranial hemorrhage. A computed tomography angiogram of the head and neck did not demonstrate large vessel occlusion. Magnetic resonance imaging of the brain revealed multiple areas of cerebral infarction (Figure 1). A spiral chest computed tomography and lung perfusion study were negative for pulmonary embolism or lung parenchymal disease. Holter monitoring for 48 hours showed no evidence of atrial arrythmia. The patient was seen by the outpatient neurology team post-discharge who recommended 30-day event monitoring if the patient experienced any new lightheadedness or palpitations. Serum homocysteine was normal, and a bilateral lower extremity duplex interrogation was negative for thrombosis.

**Figure 2:**
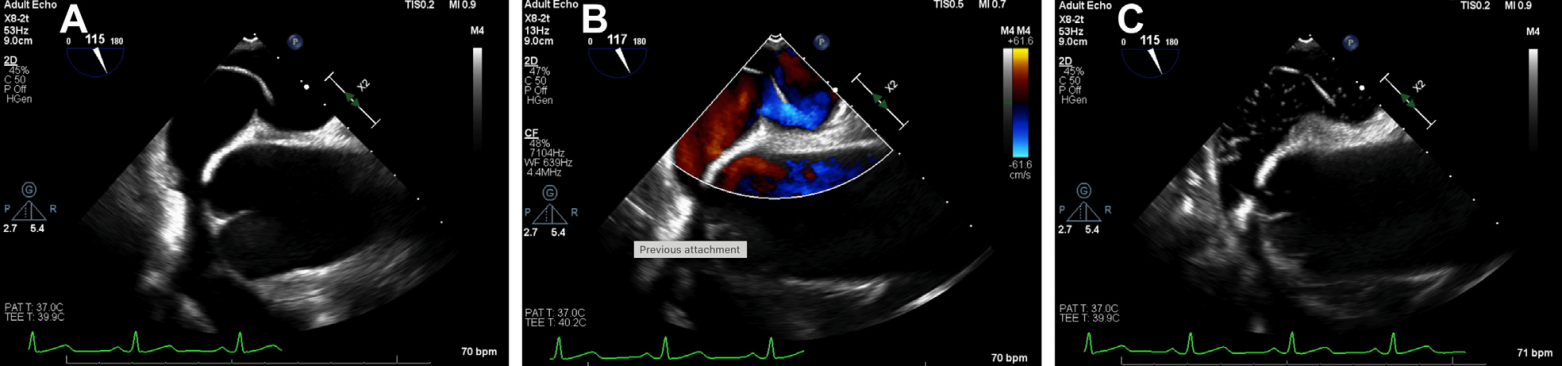
Transesophageal Echocardiography (A) Initial transesophageal echocardiography (TEE). (B) TEE with color Doppler. (C) TEE with bubble study. TEE reveals a mobile intra-atrial septum with right-to-left shunting.

A transthoracic echocardiogram (TTE) with bubble study was performed given his acute cerebrovascular event and hypoxemia, revealing normal left ventricular size and systolic function with left ventricular ejection fraction estimated at 60% to 65%. There were no wall motion abnormalities or evidence of left ventricular apical thrombus. There were normal diastolic parameters, no significant valvular abnormalities, a dilated aortic root of 4.4 cm, and normal estimated pulmonary artery systolic pressure of 24 mm Hg. Because of positionally dependent hypoxemia, TTE was performed with the patient both sitting and standing suspecting platypnea-orthodeoxia syndrome (POS). Agitated saline injection showed rapid right-to-left atrial shunting concerning for a patent foramen ovale (PFO) in the sitting position (Video 1A) and no shunting appreciated across the interatrial septum while supine (Video 1B). A transesophageal echocardiogram (TEE) was subsequently performed to delineate anatomy in preparation for PFO closure. It showed a hypermobile intra-atrial septum with a PFO measuring 0.66 cm in diameter with a positive bubble study (Video 2, Figure 2).

## Management

The patient was medically managed for a nonhemorrhagic stroke with dual antiplatelet therapy and statin medication. He continued to require high-flow nasal cannula while hospitalized.

**Figure 3:**
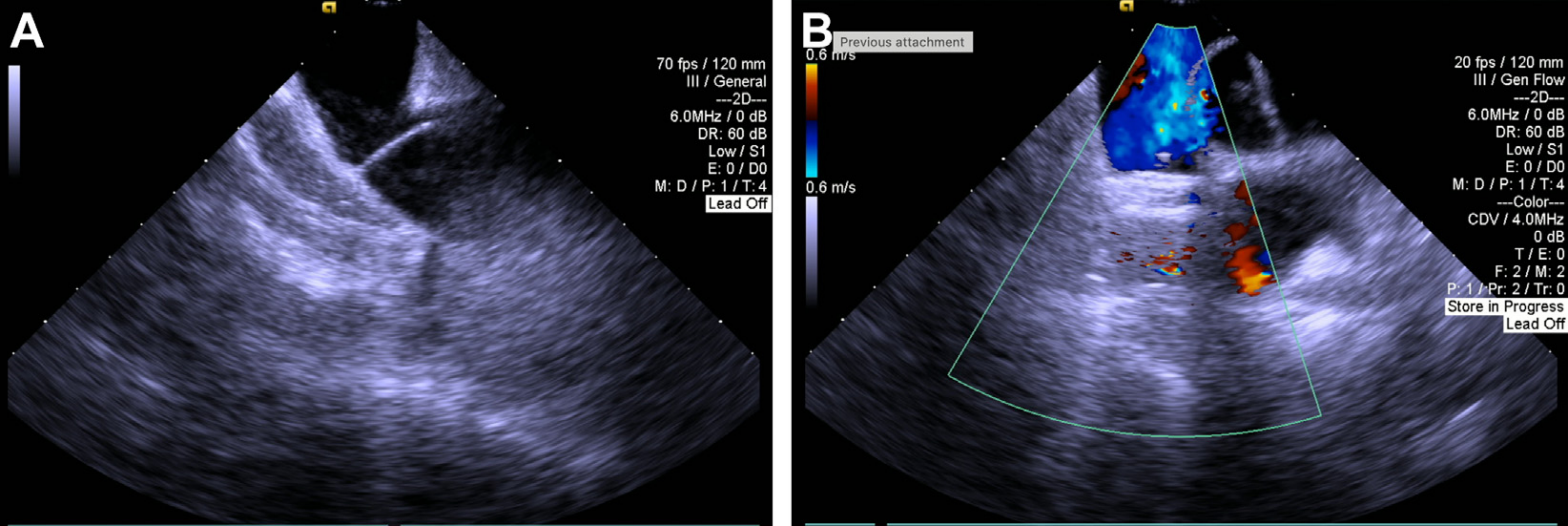
Intracardiac Echocardiography (A) Intracardiac echocardiography revealing patent foramen ovale (PFO) pre-closure. (B) Amplatzer occluder with color Doppler. Well-seated PFO closure device visualized with resolution of intra-atrial shunt.

This patient’s persistent orthodeoxia, new evidence of PFO, and right-to-left shunting (RLS) were consistent with POS, prompting interdisciplinary discussion and the decision to perform PFO closure. Intracardiac echocardiography performed during the procedure confirmed proper placement of a 25-mm Amplatzer PFO occluder and revealed no evidence of residual shunt by color Doppler interrogation (Video 3, Figure 3). The patient tolerated room air postoperatively.

## Discussion

PFO occurs in approximately 25% to 30% of the general population and commonly remains asymptomatic.^1^ Cryptogenic strokes account for 20% to 40% of ischemic strokes and must be suspected when venous thrombosis, cardioembolic cause, or large vessel source are unlikely.^2^ Other possible manifestations of PFO are migraine/vascular headaches, decompression sickness and air embolism, and POS, as appreciated in our patient.^1^

POS-related hypoxemia is positional because the interatrial communication is stretched in a standing position, resulting in increased venous return from the inferior vena cava through the defect.^3^^,^^4^ Aortic root dilation can also contribute to POS by decreasing the distance between the aorta and posterior atrial wall, thus reducing tension on the interatrial septum and leading to increased RLS.^5^^,^^6^

A diagnosis of POS requires positional dyspnea (platypnea) and a decrease in PaO_2_ >4 mm Hg or SaO_2_ >5% from supine to upright position (orthodeoxia).^7^ Definitive diagnosis is confirmed through right heart catheterization, which would confirm decreased oxygen saturation between the pulmonary vein and aorta.^7^ A TTE with bubble study showed that our patient’s PFO was the causative site of shunting. Possible etiologies of shunting span intracardiac, extracardiac, and miscellaneous etiologies with a PFO being the most reported site of an intracardiac shunt.^7^

Percutaneous PFO closure in adults with prior PFO-associated strokes is strongly recommended as opposed to antiplatelet therapy alone in patients between 18 and 60 years of age. The benefits of PFO closure in patients with a history of PFO-related stroke older than 60 years of age is less clear, and these patients may reasonably decline if procedural risks are held of higher value. The PFO-associated stroke causal likelihood (PASCAL) classification system factors a risk of paradoxical embolism (RoPE) score and functional and structural features of the PFO to estimate the risk of development of paradoxical embolism that may be useful in identifying appropriate candidates for intervention.^8^ Our patient was unique in that their clinical presentation was complicated by symptomatic POS in the absence of other significant cardiovascular risk factors. Current PFO closure guidelines support PFO closure for POS, and a multidisciplinary heart-brain discussion should be implemented. The risk-benefit ratio is ideally determined through several clinical considerations including thorough history taking, brain imaging, large vessel assessment, Holter monitoring, coagulopathy studies, echocardiography including TEE, confirmation of a PFO with delineation of its anatomy, and appropriate patient follow-up for patient engagement and education.

We add to a scarce body of literature on concomitant POS and ischemic stroke. The connection between these conditions may be partially explained by various aging-related factors, such as aortic root dilation, PFO size, kyphoscoliosis causing distortion of the interatrial septum, and stiffness of the right ventricle.^6^

## Follow-up

The patient’s facial droop and aphasia resolved while hospitalized and he has lived independently at home since the PFO closure. At a 1-month cardiology outpatient follow-up, the patient was asymptomatic and was tolerating his baseline exercise regimen. A TTE was performed 1-month post-discharge and revealed no evidence of shunting across the PFO occluder device during the bubble study and color flow Doppler interrogation. The remainder of the TTE was unremarkable with normal pulmonary arterial pressures consistent with the baseline TTE (Video 4).

## Conclusions

POS is a rare manifestation of a PFO and should be suspected in the differential diagnosis of a patient with positional dyspnea and hypoxemia. Few cases have been described regarding clinical outcomes following a repaired intracardiac shunt associated with POS due to its rarity. Our clinical case showed rapid symptomatic improvement following PFO closure and the significance of prompt intervention to reduce the risk of a paradoxical embolus.

## Funding Support and Author Disclosures

The authors have reported that they have no relationships relevant to the contents of this paper to disclose.

## Appendix

Video 1. TTE A. Bubble Study in Upright Position (A) Profound increase in right-to-left is dependent on an upright position. (B) Bubble study in supine position. There is minimal interatrial shunting.

Video 1. TTE A. Bubble Study in Upright Position (A) Profound increase in right-to-left is dependent on an upright position. (B) Bubble study in supine position. There is minimal interatrial shunting.

Video 2. Transesophageal Echocardiography A patent foramen ovale 0.66 cm in diameter is confirmed.

Video 3. Intracardiac Echocardiography With Doppler Confirming Placement of 25-mm Amplatzer Patent Foramen Ovale Occluder Device Well-seated device is visualized with immediate resolution of interatrial shunting confirmed by color flow Doppler interrogation.

Video 4. Transthoracic Echocardiography With Bubble Study Performed at 1-Month Follow-up No evidence of interatrial shunting on color flow Doppler interrogation with a well-seated interatrial device.
